# Linagliptin versus sitagliptin in patients with type 2 diabetes mellitus: a network meta-analysis of randomized clinical trials

**DOI:** 10.1186/s40199-017-0189-6

**Published:** 2017-10-25

**Authors:** Khosro Keshavarz, Farhad Lotfi, Ehsan Sanati, Mahmood Salesi, Amir Hashemi-Meshkini, Mojtaba Jafari, Mohammad M. Mojahedian, Behzad Najafi, Shekoufeh Nikfar

**Affiliations:** 1 Health Human Resources Research Center, Department of Health Economics, School of Management and Information Sciences, Shiraz University of Medical Sciences, Shiraz, IR Iran; 2 Department of Pharmacoeconomics and Pharmaceutical Administration, Faculty of Pharmacy, Tehran University of Medical Sciences, Tehran, IR Iran; 3 Atherosclerosis Research center, Baqiyatallah University of Medical Sciences, Tehran, IR Iran; 4 Iranian center of Excellence in health management, Department of health services management, School of management and medical informatics, Tabriz University of medical sciences, Tabriz, Iran; 5 Department of Pharmacoeconomics and Pharmaceutical Administration, Faculty of Pharmacy and Evidence-Based Medicine Group, Pharmaceutical Sciences Research Center, Tehran University of Medical Sciences, Tehran, Iran

**Keywords:** Linagliptin, Type 2 diabetes mellitus, Sitagliptin, Network meta-analysis

## Abstract

**Background:**

Diabetes is one of the most common chronic and costly diseases worldwide and type 2 diabetes is the most common type which accounts for about 90% of cases with diabetes. New medication-therapy regimens such as those containing linagliptin alone or in combination with other medications (within the category of DDP-4 inhibitors) must be evaluated in terms of efficacy and compared with other currently used drugs and then enter the medication list of the country. Hence, this study aimed to compare the clinical efficacy of the two drugs, i.e. linagliptin and sitagliptin, in patients with type 2 diabetes.

**Methods:**

A systematic review was conducted to identify all clinical trials published by 2015 which compared the two drugs in patients with type 2 diabetes. Using keywords such as “linagliptin”, “type 2 diabetes mellitus”, “sitagliptin” and related combinations, we searched databases including Scopus, PubMed, and Web of Science. The quality of the selected studies was evaluated using the Jadad score. Considering primary and secondary outcomes extracted from the reviewed studies, a network meta-analysis was used to conduct a systematic comparison between the two studied drugs.

**Results:**

This network meta-analysis included 32 studies (Linagliptin vs PLB: *n* = 8, Sitagliptin vs PLB: *n* = 13, Linagliptin + MET vs PLB + MET: *n* = 4, and Sitagliptin + MET vs PLB + MET: *n* = 7) and a total of 13,747 patients. The results showed no significant difference between linagliptin and sitagliptin in terms of key efficacy and safety outcomes such as HbA1c changes from baseline, body weight change from baseline, percentage of patients achieving HbA1c <7, and percentage of patients experiencing hypoglycemic events (*p* > 0.05). The results showed that the efficacy of the two drug regimens was the same.

**Conclusions:**

Based on the results, there was no significant difference between the two drugs, i.e. linagliptin and sitagliptin, in terms of efficacy; in other words, the efficacy of the two drugs was the same. Therefore, the use of these two drugs depends on their availability and cost.

**Graphical abstract:**

Graphical abstract of the network meta-analysis performed to evaluate the alternatives under the study.
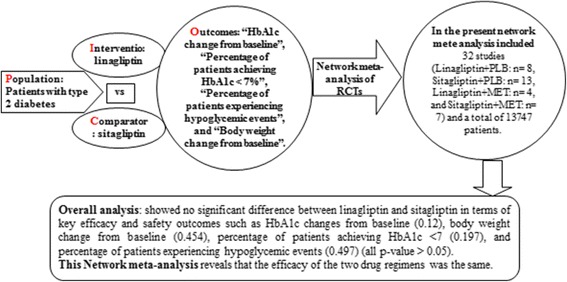

**Electronic supplementary material:**

The online version of this article (10.1186/s40199-017-0189-6) contains supplementary material, which is available to authorized users.

## Background

The incidence and prevalence of diabetes has been rapidly increasing in the last century and morbidity and mortality from these two pandemic diseases have caused massive problems for the health of human communities [1, 2]. Diabetes is one of the most common and a costly chronic disease worldwide, which is considered as a latent epidemic disease one of the health sector challenges around the world. Based on the statistical data published by the International diabetes Federation (IDF), currently 415 million people worldwide have diabetes and this number is predicted to reach 642 million people by 2040. In Iran, more than 4.6 million people were affected by the disease in 2015 [3]. Recent estimates suggest that diabetes mellitus causes 59,258,034 disability adjusted life years (DALYs) in 2012 with a 89.7% increase in deaths from diabetes since 1990 to 2013 [4].

Among different types of diabetes, type 2 diabetes is the most common type which accounts for about 90% of the cases [5]. Although the prevalence of both types of diabetes, i.e. type 1 and type 2, is increasing in the world, type 2 diabetes is more prevalent and the genetic background and environmental factors can be very effective in increasing the incidence of the disease [6–8].

Diabetes has many complications which can severely affect the quality of life of the patients and impose a high economic burden on individuals and community [9, 10].

Among available diabetes treatments, DPP4 inhibitors are one of the new generation classes with good efficacy and safety profile [11–13]. As though in a Meta- analyze study including 62 RCT studies was indicated these kind of drugs decrease HbA1c about 76% in comparison placebo [14], In other meta-analyze including 8 RCT studies, the result was shown DPP-4 decrease the risk of heart diseases in comparison MET. It seems some drugs such as linagliptin, sitagliptin and etc. can be a suitable alternatives for patients who don’t reply to MET [15].

Also, According to published clinical studies, linagliptin in this class seems to be more advantageous for patients with renal insufficiency [16]. Using evidence based approach in order to optimize resource utilization in pharmaceutical policy making has started from 2013 in Iran [17, 18]. The aim of this study was also to compare the clinical efficiency of linagliptin and sitagliptin in patients with type 2 diabetes to provide scientific evidences to policy makers for an appropriate decision making.

## Methods

### Data resources and search strategy

In order to evaluate the efficacy of the two drugs, linagliptin and sitagliptin, we conducted a systematic review of the studies published by the end of 2015. Using keywords such as “linagliptin”, “type 2 diabetes mellitus”, “sitagliptin” and related combinations, we searched databases including Scopus, PubMed, and Web of Science (Additional file 1: Appendix).

### Study selection and inclusion and exclusion criteria

Based on the inclusion criteria for this systematic review, we reviewed randomized clinical trial studies published in English that compared the clinical efficacy of linagliptin and sitagliptin. It is important to remind that these medicines had some alternatives for comparing such as placebo and metformin as shown in Tables 2 and 3. Exclusion criteria were animal studies, non-controlled trial studies, observational studies, review studies, and economic evaluation studies.

### Study selection and data extraction

PRISMA guideline was used for this systematic review, and after the initial search the duplicates were eliminated. The titles and abstracts of the remaining papers were independently evaluated by two researchers to detect and eliminate unrelated articles and those which did not meet the inclusion criteria. The results obtained by the two researchers were compared with each other and the discrepancies were resolved by referring to the articles. Then, the full-text of the selected articles was studied and the papers that met the mentioned criteria were selected.

### Quality assessment

The quality of the trials was evaluated using the Jadad score system; accordingly, each study was evaluated in terms of the three indicators, including “randomized, double blinded, and withdrawal or dropout” and scored between 0 and 5. The studies with a score more than or equal to 3 had an acceptable quality while those with a score less than 3 met the exclusion criteria. Therefore, the studies which met the intended criteria, had an acceptable quality (based on the table of Jadad score), and had the same methodology were entered into the network meta-analysis.

### Data analysis

After searching and investigating the mentioned databases, we did not find any study which directly compared the two drugs; hence, we decided to find and review clinical trials which investigated DPP-4 drugs, extract the data on their efficiency, and compare the data extracted from independent studies. Therefore, in order to integrate the results of the studies, we used network meta-analysis. To carry out the analysis, we used STATA and Excel softwares.

Thus, as noted above, in this study first we used a systematic review approach to collect studies on the efficiency of the two drugs, i.e. sitagliptin and linagliptin, which are among DPP-4 category. Then, using network meta-analysis, we analyzed the data and compared the efficacy of the drugs. In this study, we not only evaluated the efficacy of the monotherapy using each of the drugs, but also assessed the efficacy of combination therapy using the drugs together with metformin. However, due to the lack of sufficient studies, we did not assess combination therapy with other drugs, such as sulfonylureas, insulin, and glitazones.

Moreover, in order to include a study in meta-analysis, it had to meet the following PICO criteria:

P (Population): Patients with type 2 diabetes.

I (Intervention): linagliptin.

C (Comparators): sitagliptin.

O (Outcomes): The desired outcomes were: “HbA1c change from baseline”, “Percentage of patients achieving HbA1c < 7%”, “Percentage of patients experiencing hypoglycemic events”, and “Body weight change from baseline”.

To carry out this network meta-analysis, we analyzed the data using random effect approach. In this approach, first the researcher conducted a meta-analysis of the existing comparisons and combined the results via indirect meta-analysis and finally made its own comparison.

For calculating indirect effect, Bucher et al. method was used [19]. In this method the effects of lina relative to sita can be estimated indirectly as follows, using the direct estimators for the effects of PLB relative to Lina (effect_Lina,PLB_) and PLB relative to sita (effect_Sita,PLB_):

$$ {\mathrm{Effect}}_{\mathrm{Lina},\mathrm{Sita}}={\mathrm{effect}}_{\mathrm{Lina},\mathrm{PLB}}-{\mathrm{effect}}_{\mathrm{Sita},\mathrm{PLB}} $$

The indirect estimator variance of Effect_Lina,Sita_ is the sum of the direct estimators variances:

$$ {\mathrm{variance}}_{\mathrm{Lina},\mathrm{Sita}}={\mathrm{variance}}_{\mathrm{Lina},\mathrm{PLB}}+{\mathrm{variance}}_{\mathrm{Sita},\mathrm{PLB}} $$

for indirect effects of Lina + MET or Sita + MET vs PLB + MET, the same formula were used.

In this study, two tables were provided for every outcome. In the first table, it was considered that the monotherapy and combination therapy studies were not similar, while in the second table the effects of monotherapy and combination therapy were considered to be similar. For each parameter in the first table, we combined the results of the meta-analysis of the existing combinations and indirectly compared linagliptin 5 mg with sitagliptin 100 mg and also compared LIN 5 mg + MET with SIT 100 mg + MET. In the second table, it was assumed that the results of comparisons between drugs with and without MET were the same; thus, we compared linagliptin 5 mg (with and without MET) with sitagliptin 100 mg (with and without MET). For the first two outcomes, we compared the changes from the baseline and for the second two outcomes we compared the Log of odds ratios.

## Results

### Study screening, characteristics and quality of the selected studies

**Fig. 1:**
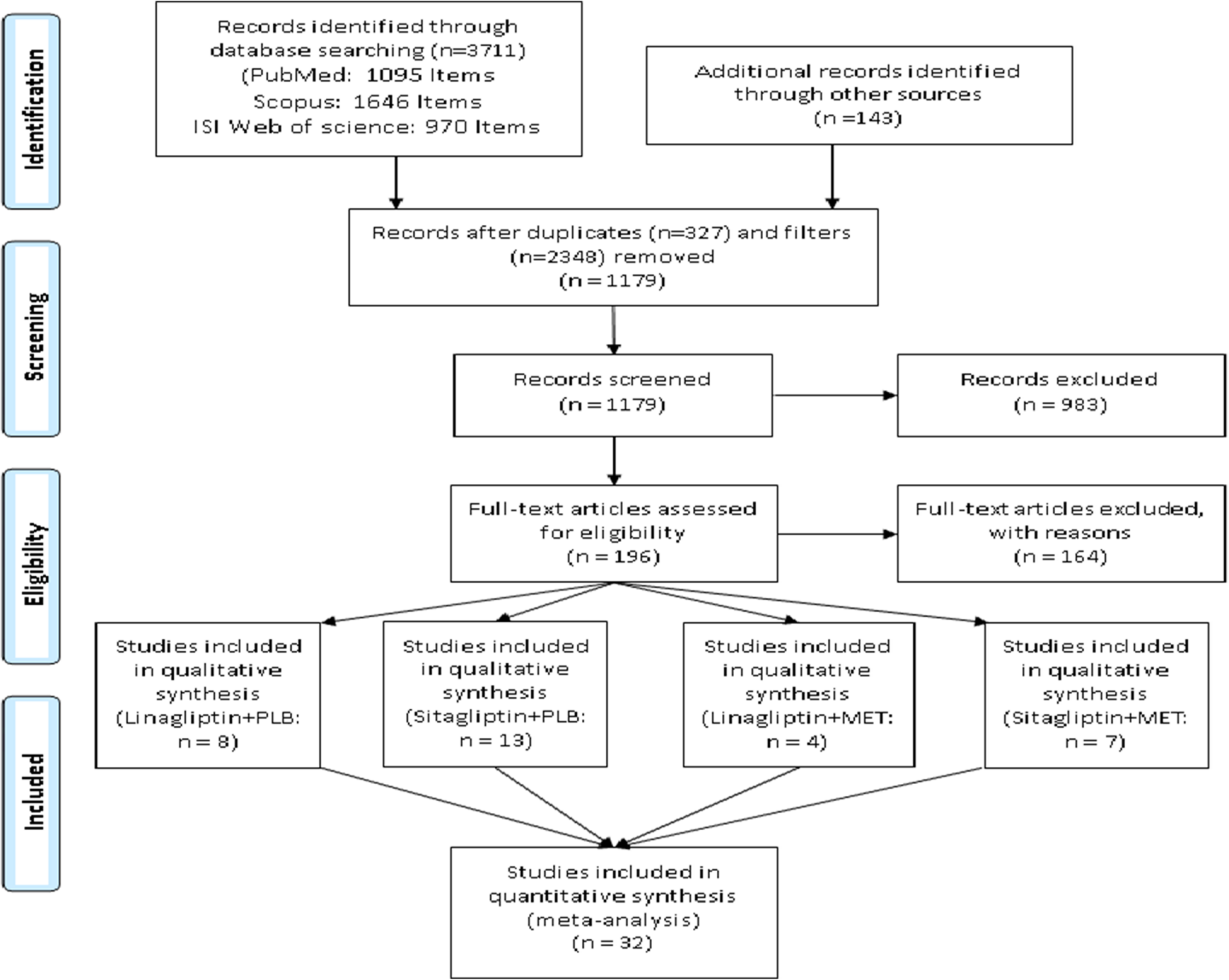
Diagram of the process of selecting clinical trials which investigated the alternatives under the study

After searching the electronic databases and reviewing the references of articles, a total of 3711 articles was obtained. All the obtained articles were carefully assessed to find the articles which met the intended criteria and finally a total of 32 articles which had an acceptable level of quality were selected for the meta-analysis. Table 1 presents the data on the selected papers (Fig. 1). It presents a summary of the characteristics of the selected studies, including the core of comparison, study period, and the number of patients. Moreover, using the Jadad score, we assessed the quality of all the selected studies; as the results showed, the all the selected studies which underwent quality assessment obtained a score equal to or more than three.

**Table 1:** Summarized characteristics of the selected studies in the network meta-analysis

Study identifier References	Treatment 1	Treatment 2	N	Weeks	Age Mean (s.d.)	Sex (males) N (%)
Linagliptin Mono. (8 RCTs)						
Del Prato et al. (2011) [31]	LIN 5 mg	PLB	503	24	55.7 (10.2)	243 (48.3)
Haak et al. (2012) [32]	LIN 5 mg	PLB	214	24	55.95 (10.9)	116 (54.2)
Kawamori (2012) [33]	LIN 5 mg	PLB	239	12	60.0 (9.1)	168 (70.2)
Barnett et al. (2012) [34]	LIN 5 mg	PLB	227	18	56.5 (10.3)	88(38.7)
Lajara (2014) [35]	LIN 5 mg	PLB	202	24	69.1(10.0)	122 (60.4)
Chen et al. (2015) [36]	LIN 5 mg	PLB	299	24	54.3 (9.7)	175 (58.5)
Taskinen (2011) [37]	LIN 5 mg	PLB	700	24	56.5 (10.3)	379 (54.1)
Inzucchi (2015) [38]	LIN 5 mg	PLB	247	24	74.3 (3.9)	126 (51.0)
Sitagliptin Mono. (13 RCTs)						
Barzilai et al. (2011) [39]	SIT 100 mg	PLB	206	24	72.0 (6.0)	97(47.1)
Nonaka et al. (2008) [40]	SIT 100 mg	PLB	151	12	55.3 (8.3)	95 (62.9)
Aschner et al. (2006) [41]	SIT 100 mg	PLB	491	24	–	–
Goldstein et al. (2007) [42]	SIT 100 mg	PLB	355	24	–	–
Hanefeld et al. (2007) [43]	SIT 100 mg	PLB	221	12	56.0 (8.5)	131 (59.2)
Scott (2008) [44]	SIT 100 mg	PLB	186	18	55.2 (9.5)	106 (57.9)
Raz (2008) [45]	SIT 100 mg	PLB	190	30	54.8 (9.5)	88(46.3)
Charbonnel (2006) [46]	SIT 100 mg	PLB	701	24	–	–
Pe’rez-Monteverde et al. (2011) [47]	SIT 100 mg	PLB	492	12	–	–
Russell-Jones et al. (2012) [48]	SIT 100 mg	PLB	409	26	54	59
Nauck 2007 [49]	SIT 100 mg	PLB	1172	52	56.7 (9.5)	694 (59.2)
Arechavaleta 2011 [50]	SIT 100 mg	PLB	1035	30	56.2 (9.9)	563(54.4)
Bergenstal (2010) [51]	SIT 100 mg	PLB	331	26	52.5 (10.5)	165 (49.8)
Linagliptin Com. (4 RCTs)						
Forst et al. (2010) [52]	LIN 5 mg + MET	PLB+ MET	137	12	59.8 (8.9)	81 (59.1)
Gallwitz et al. (2012) [53]	LIN 5 mg + MET	PLB+ MET	1551	104	59.8 (9.4)	933 (60.1)
Taskinen et al. (2011) [37]	LIN 5 mg + MET	PLB+ MET	700	24	56.5 (10.3)	379 (54.1)
Ross et al. (2012) [54]	LIN 5 mg + MET	PLB+ MET	268	12	59.1 (10.6)	142 (52.9)
Sitagliptin Com. (7 RCTs)						
Aaboe et al. (2010) [55]	SIT 100 mg + MET	PLB+ MET	24	12	59.8	17(70.8)
Arechavaleta et al. (2011) [50]	SIT 100 mg + MET	PLB+ MET	1035	30	56.2 (9.9)	563 (54.4)
Charbonnel et al. (2006) [46]	SIT 100 mg + MET	PLB+ MET	678	24	–	–
Derosa et al. (2012) [56]	SIT 100 mg + MET	PLB+ MET	178	52	55.4 (8.4)	86(48.3)
Raz et al. (2008) [45]	SIT 100 mg + MET	PLB+ MET	190	30	54.8 (9.5)	88(46.3)
Scott et al. (2008) [44]	SIT 100 mg + MET	PLB+ MET	186	18	55.2 (9.5)	106 (56.9)
Hermansen et al. (2007) [57]	SIT 100 mg + MET	PLB+ MET	229	24	57.1 (8.8)	120 (52.4)

### Outcomes

**Fig. 2:**
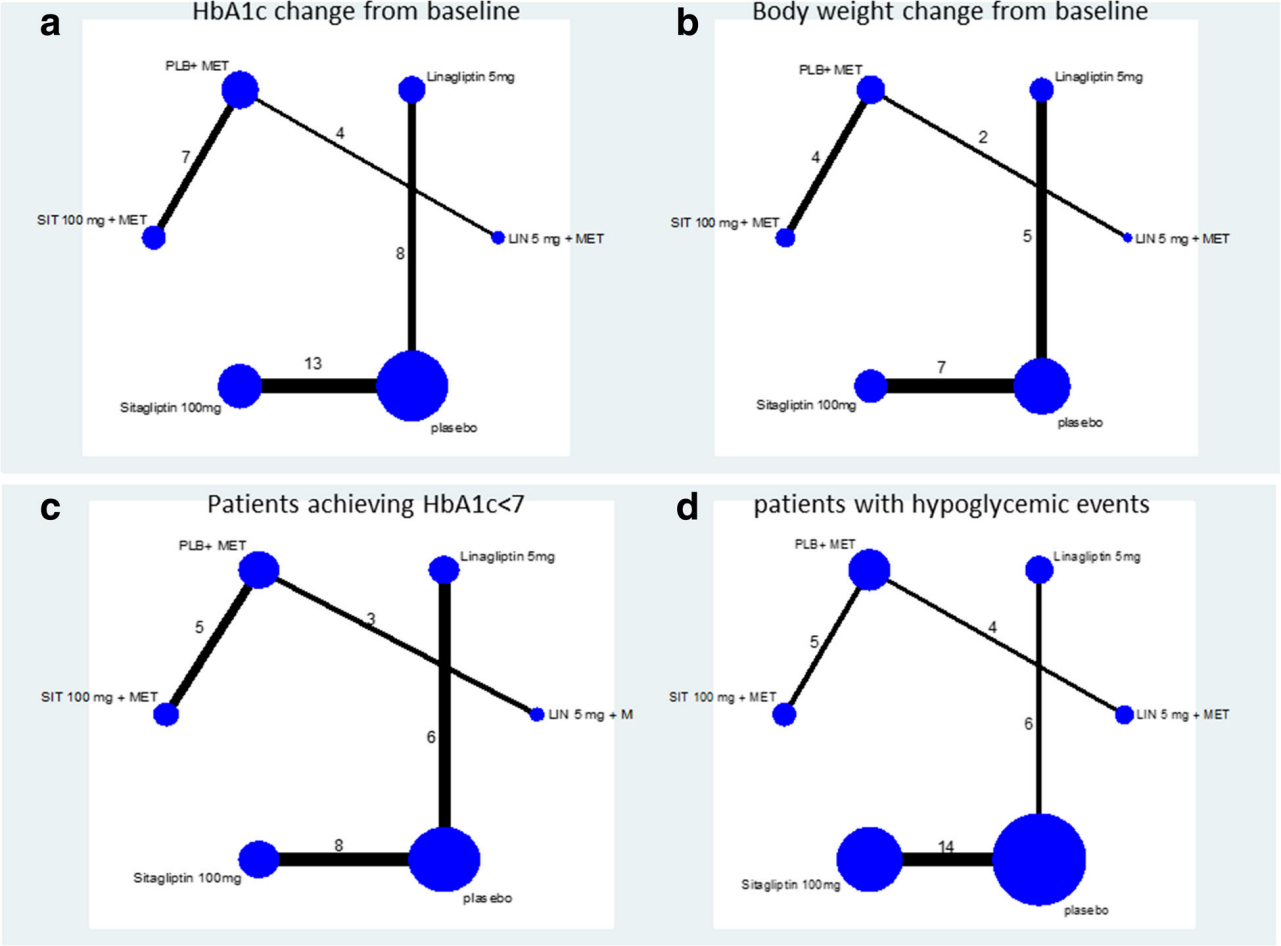
Network plot between groups

Network meta-analysis was used to compare different groups in terms of HbA1c changes from baseline, body weight change from baseline, percentage of patients achieving HbA1c <7, and percentage of patients experiencing hypoglycemic events. Figure 2 presents a schematic of the various comparisons between the groups. Figures 2a–d, respectively, show the number of studies which presented outcomes for HbA1c changes from baseline, body weight change from baseline, percentage of patients achieving HbA1c <7, and percentage of patients experiencing hypoglycemic events; these figures compare linagliptin and sitagliptin groups with the placebo group and compare LIN 5 mg + MET and SIT 100 mg + MET groups with PLB + MET group. The maximum total number of studies was 32; HbA1c changes from baseline was the outcome with the largest number of studies (32 studies) while body weight change from baseline was the outcome with the least number of studies (18 studies).

### HbA1c change from baseline

Based on the results of the meta-analysis presented in Table 2, the two groups of linagliptin and sitagliptin were significantly different from the placebo group in terms of HbA1c changes from baseline, as these two drugs, respectively, reduced HbA1c by 0.644 and 0.284 units as compared with the placebo (comparisons 1 and 2). In addition, there was no significant difference between the LIN 5 mg + MET group and PLB + MET group in terms of HbA1c changes from baseline (comparison 3). However, it showed a significant difference in SIT 100 mg + MET group, as compared with PLB + MET group, so that the drug reduced HbA1c by 0.555 units in PLB + MET group (comparison 4). Then, the results of the reported meta-analyses were combined and an indirect comparison showed a significant difference in HbA1c from baseline in linagliptin group, as compared with the sitagliptin group; in other words, linagliptin decreased HbA1c by 0.359 units, as compared with sitagliptin (comparison 5). However, the changes in LIN 5 mg + MET group, as compared with SIT 100 mg + MET group, did not show a significant difference (comparison 6).

**Table 2:** Network meta-analysis for comparison of HbA1c changes from baseline, Body weight change from baseline, Percentage of patients achieving HbA1c <7 and Percentage of patients experiencing hypoglycemic events between the 2 groups

Comparison	Drug1	Drug2	HbA1c change from baseline			Body weight change from baseline			Percentage of patients achieving HbA1c <7%			Percentage of patients experiencing hypoglycemic events		
			Freq^1^	Mean difference (SE)	*p*-value	Freq	Mean difference (SE)	*p*-value	Freq	Odds Ratio (SE)	*p*-value	Freq	Odds Ratio (SE)	*p*-value
Direct (1)	Linagliptin 5 mg	placebo	8	−0.644(.045)	0	5	0.348(.283)	0.217	6	0.712 (.153)	0	6	−0.625 (.244)	0.01
Direct (2)	Sitagliptin 100 mg	placebo	13	−0.284(.125)	0.022	7	−0.925 (.795)	0.244	8	0.440(.261)	0.091	14	−0.820(.287)	0.004
Direct (3)	LIN 5 mg + MET	PLB+ MET	4	−.247(.283)	0.383	2	−2.489 (.191)	0	3	0.924(.701)	0.188	4	−1.853 (.119)	0
Direct (4)	SIT 100 mg + MET	PLB+ MET	7	−.555(.157)	0	4	−0.201 (.839)	0.811	5	0.667 (.446)	0.135	5	−0.427(.525)	0.417
Indirect (5)	Linagliptin 5 mg	Sitagliptin 100 mg	–	−0.359(.133)	<.05	–	1.273(.843)	>0.05	–	0.272 (.302)	>.05	–	0.195 (.377)	>.05
Indirect (6)	LIN 5 mg + MET	SIT 100 mg + MET	–	0.308(.324)	>.05	–	−2.288(.860)	<0.05	–	0.257(.830)	>.05	–	−1.426(.538)	<.05

1- Freq: frequency

### Body weight change from baseline

Table 2 shows no significant difference between the linagliptin and placebo groups (comparison 1) and between sitagliptin groups and placebo group (comparison 2) in terms of body weight changes from baseline. However, it had a significant difference in LIN 5 mg + MET group, as compared with the PLB + MET group, so that the drug reduced the body weight by 2.489 units, as compared with the PLB + MET group (comparison 3). On the other hand, there was no significant difference in the SIT 100 mg + MET group, as compared with the PLB + MET group (comparison 4).

Then, the results of reported meta-analyses were combined and an indirect comparison showed no significant difference between linagliptin and sitagliptin groups in terms of body weight changes from baseline (comparison 5). However, it showed a significant difference in the LIN 5 mg + MET group as compared with the SIT 100 mg + MET groups, so that LIN 5 mg + MET decreased body weight by 2.288 units, as compared with SIT 100 mg + MET (comparison 6).

### Percentage of patients achieving HbA1c <7

As shown in Table 2, the percentage of patients achieving HbA1c <7 significantly increased in the linagliptin group, as compared with the placebo group; the odds ratio of HbA1c <7 in this group was 2.04 times more than that in the placebo group (comparison 1). However, it did not show a significant difference in the sitagliptin group, as compared with the placebo group (comparison 2). In addition, comparing the percentage of HbA1c <7 showed that there was no significant difference between the LIN 5 mg + MET group and PLB + MET group (comparison 3) and between the SIT 100 mg + MET group and PLB + MET group (comparison 4). Then, the results of reported meta-analyses were combined and an indirect comparison of the percentage of HbA1c <7 showed no significant difference between the linagliptin group and sitagliptin group (comparison 5) and between the LIN 5 mg + MET group and SIT 100 mg + MET group (comparison 6).

### Percentage of patients experiencing hypoglycemic events

As shown in Table 2, the percentage of patients experiencing hypoglycemic events significantly decreased in the linagliptin and sitagliptin groups, as compared with the placebo group; the odds ratio of hypoglycemic events in the two groups was 0.53 and 0.44, respectively, as compared with the placebo group (comparisons 1 and 2). Moreover, the percentage of hypoglycemic events significantly decreased in the LIN 5 mg + MET group, as compared with the PLB + MET group; the odds ratio of hypoglycemic events in the LIN 5 mg + MET group was OR = exp.(−1.853) = 0.15 times more than that in the PLB + MET group (comparison 3). However, there was no significant difference between the SIT 100 mg + MET group and PLB + MET group (comparison 4). Then, the results of reported meta-analyses were combined and an indirect comparison of hypoglycemic events showed no significant difference between the linagliptin group and sitagliptin group (comparison 5). However, it significantly decreased in the LIN 5 mg + MET group, as compared with the SIT 100 mg + MET group; the odds ratio of hypoglycemic events in the LIN 5 mg + MET group was OR = exp.(−1.426) = 0.24 times more than that in the SIT 100 mg + MET group (comparison 6).

As shown in Table 3, considering the similarities between comparisons 1 and 3 and between comparisons 2 and 4, there was a significant difference in HbA1c changes from baseline in the linagliptin and sitagliptin groups (with and without MET), as compared with the placebo group; the mentioned drugs, respectively, reduced HbA1c by 0.495 and 0.375 units, as compared with the placebo group (comparisons 7 and 8). However, finally, there was no significant difference between the linagliptin group (with and without MET), as compared with the sitagliptin group (with and without MET) (comparison 9).

**Table 3:** Network meta-analysis for comparison HbA1c changes from baseline, Body weight change from baseline, Percentage of patients achieving HbA1c <7 and Percentage of patients experiencing hypoglycemic events between 2 groups (If in Table 2 pairs (1 similar 3) & (2 similar 4))

Comparison	Drug1	Drug2	HbA1c change from baseline			Body weight change from baseline			Percentage of patients achieving HbA1c <7%			Percentage of patients experiencing hypoglycemic events		
			Freq	Mean difference (SE)	*p*-value	Freq	Mean difference (SE)	*p*-value	Freq	Ln(OR) (SE)	*p*-value	Freq	Ln(OR) (SE)	*p*-value
Direct (7)	Linagliptin 5 mg	placebo	12	−.495(.119)	0	7	−0.211(.701)	0.764	9	0.711 (.257)	0.006	10	−1.250 (.271)	0
Direct (8)	Sitagliptin 100 mg	placebo	20	−.375(.072)	0	11	−0.664 (.553)	0.229	13	0.514 (.209)	0.014	19	−0.753 (.228)	0.001
Indirect (9)	Linagliptin 5 mg	Sitagliptin 100 mg	–	−0.12(.139)	>.05	–	0.454 (.893)	>0.05	–	0.197 (.332)	>.05	4	−0.497(.354)	>0.05

Comparing the groups in terms of body weight changes from baseline showed that there was no significant difference between the linagliptin and sitagliptin groups (with and without MET), as compared with the placebo group (with and without MET) (comparisons 7 and 8). In addition, finally, there was no significant difference between the linagliptin group (with and without MET), as compared with the sitagliptin group (with and without MET) in terms of body weight changes from baseline (comparison 9).

Comparing the groups in terms of the percentage of HbA1c <7 revealed that there was a significant difference between the linagliptin and sitagliptin groups (with and without MET), as compared with the placebo group (with and without MET) as the odds ratio of HbA1c in the linagliptin and sitagliptin groups, respectively, was 2.03 and 1.67, as compared with the placebo group (comparisons 7 and 8). However, finally, there was no significant difference between the linagliptin group (with and without MET), as compared with the sitagliptin group (with and without MET) (comparison 9).

Comparing the groups in terms of hypoglycemic events showed that there was a significant difference between the linagliptin and sitagliptin groups (with and without MET), as compared with the placebo group (with and without MET) as the odds ratio of hypoglycemic events in the linagliptin and sitagliptin groups, respectively, was 0.28 and 0.47, as compared with the placebo group (comparisons 7 and 8). However, there was no significant difference between the linagliptin group (with and without MET), as compared with the sitagliptin group (with and without MET) (comparison 9).

## Discussion

Using meta-analyses approach, we performed a direct-comparison between the drugs and placebo that the results were different. The results of the meta-analysis showed a significant difference in HbA1c changes from baseline in the linagliptin group and sitagliptin group (with and without MET), as compared with the placebo group, because HbA1c was reduced by these drugs, as compared with the placebo. It is in the same line with the results of the study of Gross JL et al. [20] However, there was no significant difference in the LIN 5 mg + MET group, as compared with the PLB + MET group.

Considering body weight changes from baseline revealed that there was no significant difference between the linagliptin and placebo groups and between the sitagliptin groups (with and without MET) and placebo group. However, it showed a significant difference in the LIN 5 mg + MET group, as compared with the PLB + MET group, so that the drug reduced body weight, compared with the PLB + MET group.

As to the percentage of patients achieving HbA1c <7, it significantly increased in the linagliptin group, as compared with the placebo group; the odds ratio of HbA1c <7 in this group was two times more than that of the placebo group. However, there was no significant difference in the LIN 5 mg + MET and sitagliptin groups (with and without MET), as compared with the placebo group. Moreover, as to the percentage of hypoglycemic events, there was a significant difference in the linagliptin (with and without MET) and sitagliptin groups, as compared with the placebo group. The odds ratio of hypoglycemic events in the two groups was less than that in the placebo group. Nevertheless, there was no significant difference in the SIT 100 mg + MET group, as compared with PLB + MET group.

The results of network meta-analysis showed no significant difference in HbA1c changes from baseline, body weight changes from baseline, percentage of HbA1c <7, and percentage of hypoglycemic events in the linagliptin group (with and without MET), as compared with the sitagliptin group (with and without MET); thus, the efficiency of the two drugs are identical. Therefore, the results of this study are consistent with those of the studies conducted in other countries [20–22].

As noted in the results, in certain cases linagliptin has a relative advantage in pharmacokinetic superiority over sitagliptin; thus, it can be considered as a suitable alternative. Non-renal clearance is one of the pharmacokinetic features of linagliptin which makes it different from other gliptins available on the market. Therefore, its use in patients with renal insufficiency is safe and does not have any restriction [16]. Therefore, this drug is more advantageous, as compared with other drugs in this category, and is considered to be the treatment of choice in patients with renal insufficiency because kidney dysfunction is quite common in diabetic patients [23–26].

Although the efficacy of linagliptin and sitagliptin is the same, because the use of linagliptin has no restrictions in patients with renal dysfunction, and this drug is a better alternative for patients with renal dysfunction, it is necessary to make it accessible in Iran pharmaceutical market along with sitagliptin so that the physicians have an opportunity to prescribe thesedrugs for patients with different background diseases.

The results of meta-analysis can be different from those of indirect comparisons; however, we should be more cautious when interpreting the results of indirect comparisons. Systematic reviews and evaluation of the quality of clinical trials can help reduce the errors in the results of network meta-analysis but when there is a difference between the results of direct-comparison meta-analysis and indirect meta-analysis, it is necessary to re-examine the validity and generalizability of clinical trials to find the reason for the error. On the other hand, recent experimental results indicate the match between these two types of comparisons [27]. When using studies with a low quality, the results of indirect-comparison meta-analysis may be incomplete [28, 29]; on the other hand, it might be considered incomplete due to inherent differences caused via choosing the right plan, or due to limitations in comparing all the items one by one [30]. However, in this study, all the studies were examined in detail by expert people and they underwent quality assessment. At the end, this study compared linagliptin and sitagliptin drugs in terms of main efficacy and safety outcomes mentioned above. The results of this study showed no significant difference between linagliptin and sitagliptin in terms of clinical efficiency. Therefore, given their similar level of efficacy, the use of these two drugs depends on their availability and cost. According to this fact that efficacy of Linagliptin and sitagliptin is not statistically different in terms of main outcomes, any recommendation for use of each of them could be only based on cost and renal functionality of patients. For patients with renal impairments who cannot use sitagliptin and are preferred to use DPP4 inhibitors, linagliptin is a good choice. But for other patients, an economic study should be performed on the results of this study to consider economic perspective in decision making.

## Conclusion

The results showed no significant difference between linagliptin and sitagliptin in terms of clinical efficacy and they had the same effect. However, as there is no restriction on the use of linagliptin in patients with renal dysfunction, it might be considered as a treatment of choice. Hence, it is recommended to include this drug, along with sitagliptin, in the list of pharmaceuticals in Iran.

## Additional file

Additional file 1: Appendix. Search strategies. (DOC 215 kb)

## Abbreviations

DALYs: Disability adjusted life years
DPP- 4: Dipeptidyl peptidase-4 inhibitors
IDF: International diabetes Federation
LIN: Linagliptin
MET: Metformin
OR: Odds Ratio
PICO: Population; Intervention; Comparators; Outcomes
PLB: Placebo
SIT: Sitagliptin
