# Supplementary material for: Linagliptin versus sitagliptin in patients with type 2 diabetes mellitus: a network meta-analysis of randomized clinical trials
Source: Daru. 2017 Oct 25;25:23. doi: 10.1186/s40199-017-0189-6 (PMC5655990; doi:10.1186/s40199-017-0189-6)
Supplement: Additional file 1: — Appendix. Search strategies. (DOC 215 kb) [file 40199_2017_189_MOESM1_ESM.doc]

**Appendix:**

Search strategies and search results for clinical trials in databases

| Table1. Search results in PubMed | | |
| --- | --- | --- |
| Result | Regulation | Search |
| 359 items | (Linagliptin OR bi-1356 OR OR bi 1356 OR bi1356 OR ondero) | #1 |
| 1428 Items | (sitagliptin OR km-0431 OR km 0431 OR km0431 OR januvia) | #2 |
| 1678 Items | #1 OR #2 | #3 |
| 108644 Items | type 2 diabetes mellitus | #4 |
| 1095 Items | #3 AND #4 | #5 |
| 280 Items | Filters: Clinical Trial; Publication date to 2015/12/31; Humans; English | Inclusion criteria |

| Table2. Search results in Scopus | | |
| --- | --- | --- |
| Result | Regulation | search |
| 3918 Items | ALL ( linagliptin  OR  bi-1356  OR  bi  1356  OR  ondero ) | #1 |
| 1358 Items | ALL ( sitagliptin  OR  km-0431  OR  km  0431  OR  januvia ) | #2 |
| 457264 Items | ALL ( type  2  diabetes  mellitus ) | #3 |
| 1646 Items | (#1 OR #2) AND #3 | #4 |
| 461 Items | (REFPUBYEAR > 1990 ) ( LIMIT-TO ( DOCTYPE ,  "ar" ) )  AND  ( LIMIT-TO ( EXACTKEYWORD ,  "Human" ) )  AND  ( LIMIT-TO ( LANGUAGE ,  "English" ) ) | Inclusion criteria |

| Table3. Search results in Web of science | | |
| --- | --- | --- |
| Result | Regulation | search |
| 515 Items | TS=((linagliptin) OR (bi-1356) OR (bi 1356) OR (ondero)) | #1 |
| 2108 Items | TS=((sitagliptin) OR (km-0431) OR (km 0431) OR (januvia)) | #2 |
| 71431 Items | type 2 diabetes mellitus | #3 |
| 2492 Items | #1 OR #2 | #4 |
| 970 Items | #3 AND #4 | #5 |
| 646 Items | Refined by: LANGUAGE: (English) AND DOCUMENT TYPES: (Article) | Inclusion criteria |

The tables showing the results of analyses are presented below. The numbers 0, 1, 2, and 3, respectively, represent studies on linagliptin monotherapy, sitagliptin monotherapy, linagliptin and metformin combination therapy, and sitagliptin and metformin combination therapy. The orders of the studies are similar for all the four studied efficacy indicators.

Comparison of HbA1cchanges from baseline

Comparison of Body weight change from baseline

Comparison of the Percentage of patients achieving HbA1c <7

Comparison of the Percentage of patients experiencing hypoglycemic
